# Novel Borna Virus in Psittacine Birds with Proventricular Dilatation Disease

**DOI:** 10.3201/eid1412.080984

**Published:** 2008-12

**Authors:** Kirsi S. Honkavuori, H.L. Shivaprasad, Brent L. Williams, Phenix-Lan Quan, Mady Hornig, Craig Street, Gustavo Palacios, Stephen K. Hutchison, Monique Franca, Michael Egholm, Thomas Briese, W. Ian Lipkin

**Affiliations:** Columbia University, New York, NY, USA (K.S. Honkavuori, B.L. Williams, P.-L. Quan, M. Hornig, C. Street, G. Palacios, T. Briese, W.I. Lipkin); University of California Animal Health and Food Safety Laboratory System–Fresno Branch, Davis, California, USA (H.L. Shivaprasad, M. Franca); 454 Life Sciences, Branford, Connecticut, USA (S.K. Hutchison, M. Egholm)

**Keywords:** Borna disease virus, birds, proventricular dilatation disease, real time PCR, high throughput pyrosequencing, dispatch

## Abstract

Pyrosequencing of cDNA from brains of parrots with proventricular dilatation disease (PDD), an unexplained fatal inflammatory central, autonomic, and peripheral nervous system disease, showed 2 strains of a novel Borna virus. Real-time PCR confirmed virus presence in brain, proventriculus, and adrenal gland of 3 birds with PDD but not in 4 unaffected birds.

Borna disease virus (BDV) is the causative agent of Borna disease, a meningoencephalitis of horses and sheep in central Europe (*1*). As the prototype and only known member of the family *Bornaviridae* in the order Mononegavirales (nonsegmented, negative-strand RNA viruses), BDV is atypical in its nuclear localization of transcription, alternative splicing, and differential use of initiation and termination signals. Sequence analysis of isolates obtained from various species over several decades has shown remarkable sequence conservation; only 2 genotypes are known. The virus is highly neurotropic and infects the central, peripheral, and autonomic nervous systems. Although ungulates remain the best known natural host, the introduction of sensitive molecular and serologic assays enabled by subtractive cloning of the BDV genome facilitated surveys that indicated wider geographic and species distribution (*1*). Experimental infections are described in a wide variety of vertebrates including chickens, quails, rats, rabbits, cats, shrews, and nonhuman primates; manifestations of disease range from fatal meningoencephalitis to subtle behavioral alterations or asymptomatic persistent infection (*2*). Intestinal colic is frequently observed in infected ungulates (*2*,*3*). An outbreak of neurologic disease in farmed ostriches in Israel has been attributed to BDV (*4*). BDV nucleic acids have been reported in feces of wild mallards and jackdaws in Sweden (*5*).

Proventricular dilatation disease (PDD), also known as proventricular dilatation syndrome or macaw wasting disease, is a disorder of birds wherein inflammation of the central, peripheral, and autonomic nervous systems is associated with gastrointestinal dysfunction and neurologic signs that may include ataxia and seizures (*6*). Although a presumptive diagnosis can be achieved through imaging studies and biopsy, for most animals, definitive diagnosis is made postmortem, only after detailed histologic analysis indicates the accumulation of lymphocytes in nerves that supply the proventriculus and ventriculus, in their associated ganglia, and in brain. Features consistent with PDD have been reported in >50 avian species; however, PDD is most commonly described in exotic companion birds such as macaws and parrots. Whether this reflects better case ascertainment or factors that influence exposure or susceptibility is unclear. An infectious basis is supported by the observation that disease can be transferred to naive birds through inoculation with tissue homogenates or fecal material from affected birds (*7*). One electron microscopic study showed the presence of spherical, 83-nm particles in macaw embryo cells after inoculation with feces from a diseased macaw; other researchers have described particles in tissue consistent in appearance with adenoviruses or paramyxoviruses. Whether any of these agents can be implicated in the pathogenesis of PDD is unknown.

On a quest for the causative agent of PDD, we investigated 3 birds with a PDD diagnosis based on clinical history and histologic criteria (Table 1). RNA was extracted from brains, pooled and randomly amplified for unbiased high-throughput sequencing (*8*), yielding 96,698 reads, ranging from 40 nt to 353 nt. After implementation of algorithms for vertebrate sequence subtraction and contiguous fragment assembly, GenBank searches using BLAST (http://blast.ncbi.nlm.nih.gov/blast.cgi) indicated a relation to BDV for a total of 11 contigs covering ≈1.1 kb of sequence distributed in 6 clusters throughout the N (230 nt), P (450 nt), G (250 nt), and L (120, 80, and 250 nt) genes (Figure 1). Divergent sequences between multiple overlapping contigs in these 6 regions indicated at least 2 different strains. Analysis of ≈5.5 kb of genomic sequence generated by standard PCR by using primers (sequence available upon request) based on the identified sequence fragments confirmed 2 strains, one from bird 1367, another from birds 1034 and 1322 (GenBank accession nos. FJ169440 and FJ169441, respectively) and indicated conservation of the unique genome organization that is characteristic for the family *Bornaviridae* (Figure 1). Sequence divergence between the avian strains (86% identity at nucleotide level) is similar to that observed between the most divergent strains isolated from ungulates (84% identity). Pairwise comparison of the avian strains with these 2 ungulate isolates that represent the 2 previously known genotypes of BDV, strain V, NC_001607 (*9*), and No/98, AJ311524 (*10*), indicated <70% sequence conservation at the nucleotide level and <80% at the overall amino acid level (Figure 2, Table 2). These data are compatible with the avian strains representing a new species.

**Table 1:** Real-time PCR measurement of viral sequences in birds with PPD*

Case no.	Species†	Age/sex	PDD/ control	Primer/probe set	Organ	Ct	Virus copies‡ (300 ng total RNA)
1034	Canindae macaw	30 y/M	PDD	1034–1322	Brain	17.94	8.88 × 10^7^
					Adrenal gland	20.51	8.77 × 10^6^
					Proventriculus and gizzard	25.26	1.22 × 10^5^
1322	Vinaceous Amazon	1 y/M	PDD	1034–1322	Brain	17.64	1.17 × 10^8^
					Proventriculus	28.47	6.76 × 10^3^
1367	Canindae macaw	30 y/F	PDD	1367	Brain	27.33	8.89 × 10^3^
					Adrenal gland	18.92	3.87 × 10^6^
					Proventriculus	23.82	1.13 × 10^5^
5473	Leadbetter’s cockatoo	17 y/M	Control	1034–1322 and 1367	Brain	>36§	Negative
4858	Long-tailed parakeet	Adult/M	Control	1034–1322 and 1367	Brain	>36§	Negative
2020	Cockatiel	Young/M	Control	1034–1322 and 1367	Brain	>36§	Negative
3616	Eclectus parrot	0.25 y/F	Control	1034–1322 and 1367	Brain	>36§	Negative

*PPD, poventricular dilatation disease; Ct, cycle threshold. †All birds were captive animals from California, USA. ‡Copy numbers were calculated on the basis of a standard curve generated from cloned target sequences. §A Ct of >36 was rated as negative based on the highest dilution of standard representing 5 copies.

**Figure 1:**
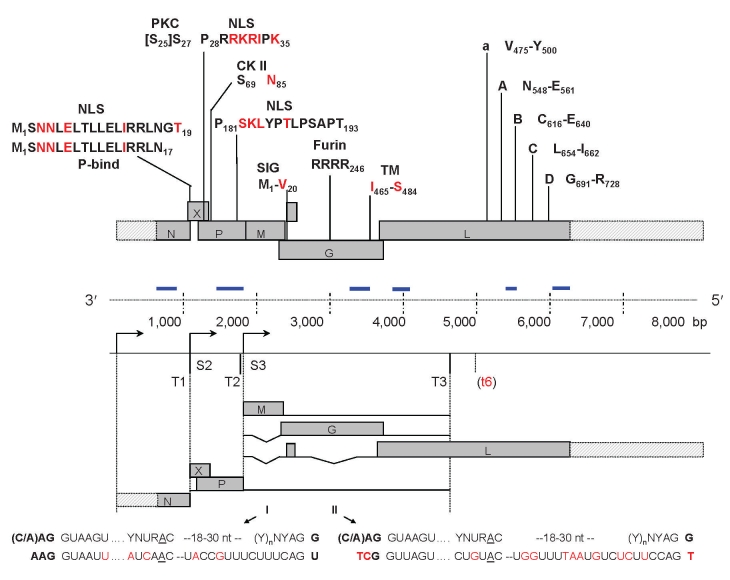
Conservation of genome organization, regulatory sequences, and protein domains of Borna disease virus (BDV) in novel strains from parrots 1034, 1322, and 1367. N, nucleoprotein; P, phosphoprotein; X, X protein; M, matrix protein; G, glycoprotein; L, L-polymerase protein. Genome regions not yet sequenced in the novel strains are shaded. P-bind, binding site for P on X; NLS, nuclear localization signals of X and P; PKC, protein kinase C epsilon phosphorylation sites in P; CK II, casein kinase phosphorylation sites in P; SIG, signal peptide; Furin, furin cleavage site; TM, transmembrane anchor of G; A – D, conserved RNA-dependent RNA polymerase motifs. Conserved sites/residues with respect to BDV strain V are shown in black; divergent sites/residues are indicated in red; K_32_ in P NLS-1 is divergent only in 1034/1322, K_35_ in NLS-1 and K_183_ in NLS-2 are divergent only in 1367. S2 and S3, start sites of transcription units 2 and 3, respectively, showing the conserved GAA initiation triplet; T1, T2, and T3, transcription termination sites showing the conserved TA_6_ consensus sequence; (t6) indicates a nonconserved TA_6_ sequence found in some BDV isolates. Blue bars indicate the 6 clusters represented by contigs obtained through pyrosequencing. Consensus splice site sequences corresponding to established introns I and II in genes for M and G of BDV strain V are aligned to corresponding sequences of the novel strains.

**Figure 2:**
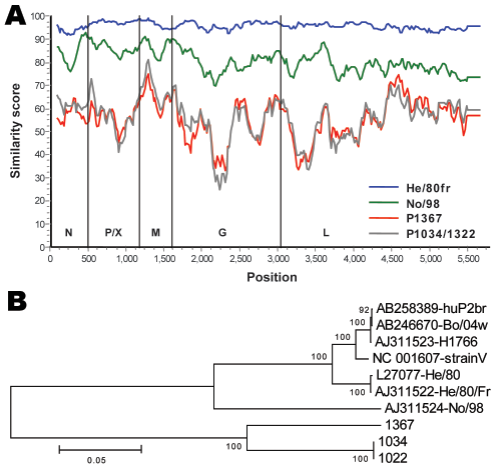
A) Similarity plot between Borna disease virus (BDV) prototype strain V nucleotide sequence and those of characterized BDV strains He/80fr and No/98 compared with novel strains 1367 and 1034/1322. Gene regions corresponding to the nucleoprotein (N), phosphoprotein (P), X protein (X), matrix protein (M), glycoprotein (G) and L-polymerase protein (L), and nucleotide positions are indicated. B) A tree representing the evolutionary history was inferred by using the neighbor-joining method. The percentage of replicate trees in which the associated taxa clustered together in the bootstrap test (500 replicates) is shown next to the branches. The tree is drawn to scale with branch lengths in the same units as those of the evolutionary distances used to infer the phylogenetic tree (number of base substitutions per site; see scale bar). Evolutionary distances were computed by using a Kimura 2-parameter model; a total of 5,449 positions in the final dataset were analyzed by using MEGA4 software (www.megasoftware.net).

**Table 2:** Percent sequence conservation between Borna disease virus strain V(No/98) and novel strains

Amino acid	Nucleotide			
	Strain V	No/98	1034/1322	1367
Strain V		84	67	66
No/98	P: 99/96*			
	X: 84/81			
	M: 100/98		66	66
	G: 95/94			
	L: 98/96			
1034/1322	P: 76/61	P: 77/63		
	X: 55/51	X: 49/45		
	M: 91/85	M: 91/85		86
	G: 75/66	G: 75/66		
	L: 81/74	L: 81/74		
1367	P: 75/60	P: 76/63	P: 98/95	
	X: 59/53	X: 53/48	X: 88/88	
	M: 91/82	M: 91/81	M: 99/95	
	G: 74/66	G: 74/66	G: 94/93	
	L: 82/76	L: 82/76	L: 97/95	

*Amino acid conservation is indicated as percent similarity/percent identity.

Primers and probes for quantitative real-time PCR were selected in the amino-terminal region of the phosphoprotein (P) gene matching P sequences for strain 1367 (set 1367, forward: 5′-AGAAGACCCGCTGACAGCA-3′, reverse: 5′-AAGCTTCTCGACGGGAACAG-3′, probe: 6FAM-5′-TCGTGGGGACCTCGATCTCACTCG-3′-TMR) or strain 1034/1322 (set 1034–1322, forward: 5′-CAGACAGCACGTCGAGTGAGA-3′, reverse: 5′-AGTTAGGGCCTCCCTGGGTAT-3′, probe: 6FAM-5′-AGGTCCCCGCGAAGGAAGCGA-3′-TMR); the diagnostic assay has been made available through ProMed mail (www.promedmail.org, archive no. 20080726.2287). Real-time PCR showed levels of viral RNA exceeding 10^3^ copies in all tissues tested from birds with PDD but not in control birds. In 2 birds (1034 and 1322) virus load was higher in the brain than in the proventriculus and gizzard or adrenal gland; in 1 bird (1367), the load was lower in the brain than in the adrenals or proventriculus (Table 1).

Western immunoblot and nondenaturing dot blot experiments were pursued by using brain, proventriculus, and adrenal homogenates of the 3 animals that were quantitated by real-time PCR, and 2 rabbit polyclonal antibodies raised against recombinant BDV strain V nucleoprotein (N) or P, as well as immune sera from BDV He/80–infected rats. Positive and negative controls were BDV He/80–infected rat brain homogenate and uninfected rat brain homogenate, respectively. A strong signal was obtained with positive control material in western immunoblots and dot blots that used either antisera to recombinant viral proteins or rat immune sera; no signal was obtained with bird homogenates or uninfected rat brain homogenate.

## Conclusions

We have not yet determined whether purified virus induces PDD or an adaptive immune response occurs in association with disease. Nonetheless, given what is known about BDV pathogenesis with other strains in other hosts (*11*), the bornaviruses identified in these birds must be considered a biologically plausible candidate causative agent. Infection, lymphocyte infiltration, and dysfunction of the central, peripheral, and autonomic nervous system are common to PDD as well as to classical Borna disease in natural disease and experimental models (*2*,*12*,*13*). Proteins and antisera we have used for 2 decades for BDV diagnostics failed to detect this virus in our PCR-positive birds. Thus, it will be important to revisit epidemiologic surveys that we, and others, have undertaken to investigate the role of bornaviruses in human disease (*14*). From a personal perspective, we are intrigued that whereas molecular discovery of the first BDV in the late 1980s required an investment of 2 years in subtractive cloning (*15*), high-throughput sequencing, bioinformatics, and sequence databases enabled discovery of these 2 strains in 2 weeks.
